# Transcripts in the *Plasmodium* Apicoplast Undergo Cleavage at tRNAs and Editing, and Include Antisense Sequences

**DOI:** 10.1016/j.protis.2016.06.003

**Published:** 2016-08

**Authors:** R. Ellen R. Nisbet, Davy P. Kurniawan, Harrison D. Bowers, Christopher J. Howe

**Affiliations:** Department of Biochemistry, University of Cambridge, Tennis Court Road, Cambridge, CB2 1QW, United Kingdom

**Keywords:** Apicoplast, chloroplast, RNA processing, *Plasmodium*, antisense RNA, RNA editing.

## Abstract

The apicoplast, an organelle found in *Plasmodium* and many other parasitic apicomplexan species, is a remnant chloroplast that is no longer able to carry out photosynthesis. Very little is known about primary transcripts and RNA processing in the *Plasmodium* apicoplast, although processing in chloroplasts of some related organisms (chromerids and dinoflagellate algae) shows a number of unusual features, including RNA editing and the addition of 3′ poly(U) tails. Here, we show that many apicoplast transcripts are polycistronic and that there is extensive RNA processing, often involving the excision of tRNA molecules. We have identified major RNA processing sites, and have shown that these are associated with a conserved sequence motif. We provide the first evidence for the presence of RNA editing in the *Plasmodium* apicoplast, which has evolved independently from editing in dinoflagellates. We also present evidence for long, polycistronic antisense transcripts, and show that in some cases these are processed at the same sites as sense transcripts. Together, this research has significantly enhanced our understanding of the evolution of chloroplast RNA processing in the Apicomplexa and dinoflagellate algae.

## Introduction

**Figure 1:**
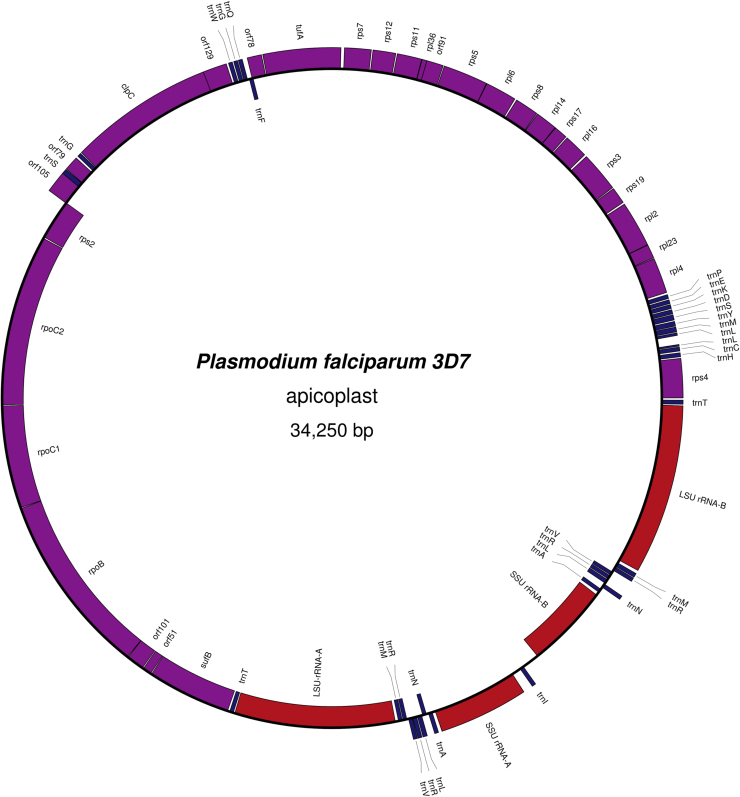
*Plasmodium falciparum* apicoplast genome. Purple indicates protein-coding genes, blue indicates tRNA genes and red indicates rRNA genes. Genome drawn using OrganellarGenomeDRAW (Lohse et al. 2013).

Some of the major drugs used for combatting malaria, such as the antibiotics doxycycline and clindamycin, target gene expression in the apicoplast, an organelle found in *Plasmodium* and other members of the Apicomplexa group of parasitic eukaryotes. The apicoplast is a secondary plastid, resulting from an endosymbiosis event between the ancestor of the Apicomplexa and a member of the red algal lineage (Botté et al., 2013, Gardner et al., 1991b, Lemgruber et al., 2013, Wilson et al., 1996). The apicoplast has lost the ability to carry out photosynthesis, yet retains a circular genome of approximately 35 kbp, containing genes for numerous proteins, tRNAs and rRNAs (Fig. 1). Inhibition of apicoplast transcription and translation is lethal to the parasite, as shown by treatment by rifampicin (a transcription inhibitor), thiostrepton or doxycycline (translation inhibitors) (Goodman et al. 2007). Inhibition of apicoplast DNA replication is also lethal (Fichera and Roos 1997).

Despite the importance of antibiotics that target the apicoplast in the control of malaria, remarkably little is known about transcription, post-transcriptional processing or translation in the organelle. Northern blots using total *Plasmodium* RNA revealed that the transcription of at least some apicoplast genes is likely to be polycistronic, as the bands seen were larger than would be expected for a single-gene RNA molecule (Gardner et al., 1991a, Gardner et al., 1991b). RT-PCR carried out on two regions indicated that some ribosomal genes were transcribed as part of a polycistronic molecule (Wilson et al. 1996), and all tRNA molecules have been shown to be transcribed (Preiser et al. 1995). There are no recognisable eubacterial promoter elements upstream of apicoplast genes, so it is unclear how transcription is initiated (Sato 2011).

The closest photosynthetic relatives of *Plasmodium* are *Chromera* and *Vitrella*, which retain fully functional chloroplasts (Janouskovec et al. 2010). We and others have recently examined transcript processing in these organisms and have shown that transcripts of many chloroplast genes are polycistronic, and those transcripts encoding proteins involved in photosynthesis are post-transcriptionally modified by the addition of a poly(U) tail at the 3′ end of the transcript. In contrast, transcripts encoding genes for proteins that are not involved in photosynthesis are generally not polyuridylylated. We have shown that *Plasmodium* apicoplast transcripts are also not polyuridylylated (Dorrell et al. 2014). Post-transcriptional processing in peridinin-containing dinoflagellates, which are a sister group to the Apicomplexa, is complex. There are a limited number of polycistronic transcripts, and these show mutually exclusive alternative cleavage pathways. Some, but not all, transcripts receive 3′ poly(U) tails. Transcripts are edited in some genera, but not all (Barbrook et al. 2012), with the ancestral state probably lacking editing.

Here, we examine in detail transcripts of the *Plasmodium* apicoplast genome, including regions that primarily encode proteins, and two regions that encode rRNAs and tRNAs. We present data showing that the genome is transcribed polycistronically, followed by cleavage to gene-specific mRNAs. Many cleavage sites are associated with tRNA sequences. Where genes are overlapping, alternative cleavage pathways occur. We also find evidence for stage-specific RNA editing. In addition, we show that there are significant levels of antisense transcripts, covering protein coding genes, tRNA and rRNA genes as well as intergenic regions. Some antisense transcripts are cleaved at the same sites as sense transcripts, suggesting that these sites may have a role in transcript processing. Given the importance of the apicoplast, these results suggest that RNA transcript processing ought to be a key target in the design of new anti-malarial drugs. The results also enhance our overall picture of the evolution of RNA processing in the apicomplexan and dinoflagellate groups.

## Results

### rRNAs are Co-transcribed with tRNA and Protein Coding Sequences

**Figure 2:**
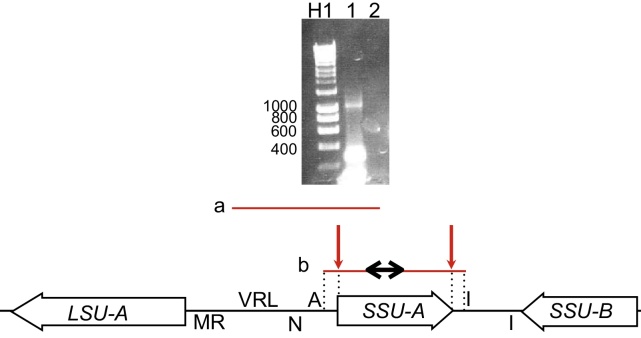
Sense transcription of LSU/SSU rRNA and tRNA locus. Arrows represent protein coding genes, letters represent tRNA genes named as per standard single-letter tRNA convention. Note that tRNAs shown above the line show genes transcribed in one strand (i.e. left to right, same orientation as the *SSU-A rRNA* gene in the middle of the figure), while those below the line show those transcribed from the other strand (i.e. right to left, same orientation as the *LSU rRNA* gene). RNA transcripts are shown in red; genomic DNA is shown in black. *Transcript a* shows the extent of linear RT-PCR products identified with a reverse primer placed within the 5′ region of *SSU-A rRNA* and forward primers at intervals towards *LSU rRNA. Transcript b* shows maximum length of sense transcripts identified by circular RT-PCR using primers within *SSU rRNA.* Red arrows show major RNA processing sites. The black arrows within the transcript indicated the region from which primers were designed. Not to scale. The agarose gel analysis of the circular RT-PCR reaction is shown above. Lane H1: Promega hyperladder 1 marker, with sizes to the left. Lane 1: RNA circularization experiment, lane 2: no reverse transcriptase control.

We began by testing if rRNA genes were represented in polycistronic transcripts, consistent with a previous report for SSU rRNA (Gardner et al. 1991a), and if polycistronic transcripts could also contain protein-coding sequences. The organization of the SSU rRNA locus, which is part of the inverted repeat region of the apicoplast genome, is shown in Figure 2. We carried out cDNA synthesis using a reverse primer within the SSU sequence, and used the cDNA and the same reverse primer in a series of PCRs with multiple forward primers located at increasing intervals upstream of the SSU rRNA gene, at least as far as the *trnV* sequence. We obtained products in all of these PCRs, indicating the existence of a transcript covering at least the whole of the region between *trnV* and the SSU rRNA (*transcript a* in Fig. 2), showing that rRNA and tRNA sequences are co-transcribed.

To map individual transcripts from SSU in more detail, we carried out RNA circularization assays targeting the SSU rRNA. In these assays, RNA is circularized using T4 RNA ligase and used for cDNA synthesis, the products of which are then analysed by PCR. Primary transcripts in organelles carry a 5′ triphosphate, while processed transcripts carry a 5′ monophosphate group (Swiatecka-Hagenbruch et al. 2007). As T4 RNA ligase catalzyes the reaction between a 5′ monophosphate group and a 3′ hydroxyl group, it can thus only calatalyse the ligation of processed RNA. Thus, these RNA circularization assays allow the identification of 3′ and 5′ ends of processed RNA transcripts.

Total RNA was circularized using an RNA ligase, and cDNA synthesized. Following RT-PCR and cloning of products, eight clones were found to contain sequence from the *SSU rRNA* region (Supplementary Material: supporting data). None of the eight transcripts clones corresponded to a polycistronic transcript, although two included the full intergenic region at both the 5′ and 3′ ends of the *SSU rRNA* gene, extending as far as, but not including, the adjacent tRNA sequences (Fig. 2, *transcript b*). The remaining six clones were derived from transcripts containing just the SSU rRNA. However, all were missing 1 bp at the 5′ end of the gene and either 6 bp (three clones) or 7 bp (three clones) at the 3′ end. These results suggest that the majority of the SSU rRNA sequences are monocistronic. In addition, the annotated 5′ and 3′ ends of the coding sequence may not be correct.

**Figure 3:**
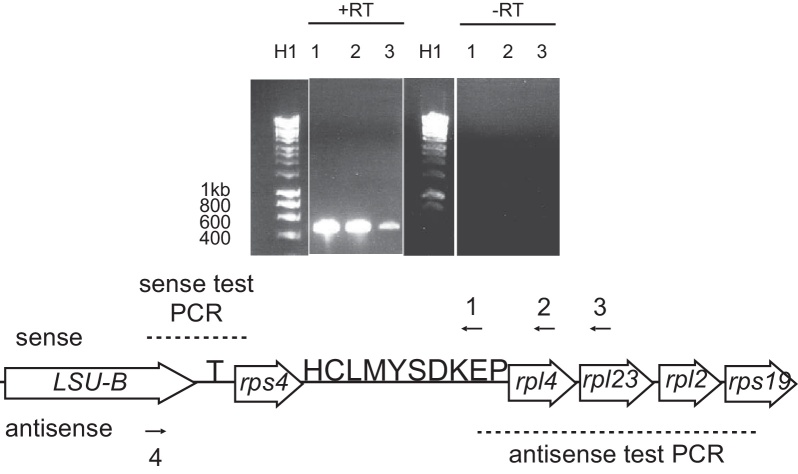
Sense and antisense transcription of the LSU/tRNA/ribosomal protein locus. Genes are shown as Figure 2. Numbered arrows 1-3 represent annealing site of primers for cDNA synthesis to identify sense transcripts, and the test sites for PCRs are labelled with black dashed lines. The associated agarose gel is shown above, with lanes corresponding to the cDNA synthesis reactions below. (Some lanes have been removed for clarity). Note that the test PCR is the same for each cDNA, and thus all PCR products should be the same length. Lane H1 is Bioline Hyperladder 1 marker, with sizes of DNA fragments indicated in bp. Arrow 4 below the genes represent the annealing site of the primer to identify antisense transcripts, and the test PCR region is shown. Although representative, the figure is not to scale.

We next tested for the presence of polycistronic transcripts from the LSU rRNA gene concentrating on the downstream region to see if transcripts could contain rRNA, protein-coding and tRNA sequences. cDNA was synthesized using a series of reverse primers (Fig. 3, primers 1-3) and the ability of the cDNA to generate PCR products using primers within the *LSU rRNA* and *rps4* genes was tested. cDNA generated from all of the reverse primers gave products in the test PCR, indicating the existence of a polycistronic RNA molecule spanning the whole region from *LSU rRNA* to *rpl23* and thus containing rRNA, tRNA and protein sequences. (Fig. 3, lanes 1-3). (Note that the test region is the same for each cDNA, and thus all PCR products should be the same size.) As the majority of LSU rRNA transcripts would be expected to contain just the rRNA sequence (as shown for the SSU rRNA), we did not perform circularized RNA assays. These results therefore show that both the SSU and LSU rRNAs are initially transcribed polycistronically, with both tRNA and protein-coding sequences.

### Transcription of Protein-coding Genes

**Figure 4:**
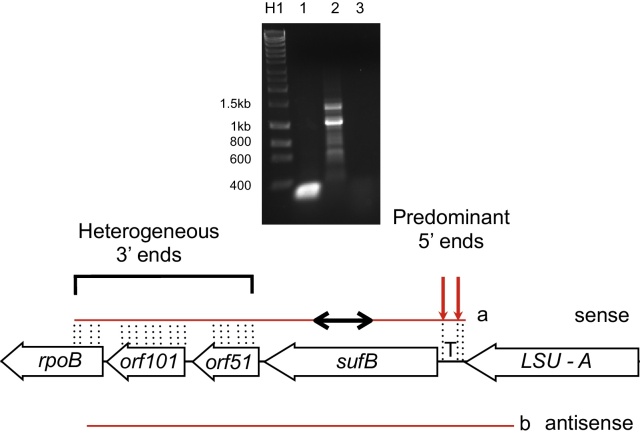
Sense and antisense transcription of *sufB* locus. Genes and transcripts are shown as Figure 2. *Transcript a* shows maximum length of sense transcripts identified by circular RT-PCR using primers within *sufB*. Arrows show major processing sites, further processing sites indicated by dotted vertical lines. The block within the transcript indicates the region from which primers were designed. *Transcript b* shows maximum extent of linear antisense RT-PCR products across the region. Not to scale. The gel shows results of circular RT-PCR for *sufB.* Lane H1, hyperladder 1 kb (Bioline) with size markers indicated in bp, lane 2, internal (control) *sufB* PCR, lane 3 *sufB* circular PCR, lane 3 no RT-control *sufB* inwards PCR.

***sufB.*** We next examined the transcripts of *sufB* (encoding an iron sulphur cluster assembly protein) using the circular RT-PCR technique (Fig. 4). Twenty four clones were obtained using *sufB* primers on cDNA from circularized RNA and sequenced (details in Supplementary Materials: supporting data S1). These revealed that the majority of transcripts (18/24) commenced 98 bp upstream of *sufB*, exactly consistent with the 5′ end of the *trnT* gene, although a substantial minority of transcripts (5/24) commenced 25 nt upstream of *sufB*, exactly consistent with the 3′ end of the adjacent *trnT* gene (Fig. 4, *transcript a* and Supplementary Material Fig. S1). The remaining transcript commenced 104 nt upstream. This would suggest that a long, initial primary transcript is created, which is cleaved at the 5′ and 3′ ends of *trnT*, releasing both the tRNA and a *sufB* mRNA with a 5′ UTR of 25 nt.

The downstream ends of the *sufB* transcripts were heterogeneous. All transcripts extended past *sufB* into the adjacent *orf51* gene, while the longest included both *orf51* and *orf101* together with a small portion of *rpoB*. Together, these results indicate that there is a transcript spanning from at least *trnT* into *rpoB* which is cleaved at a limited number of 5′ sites associated with the tRNA. The 3′ ends within protein coding sequences are much more heterogeneous, either as a result of exo-or endo-nucleolytic cleavage, or transcription termination.

**Figure 5:**
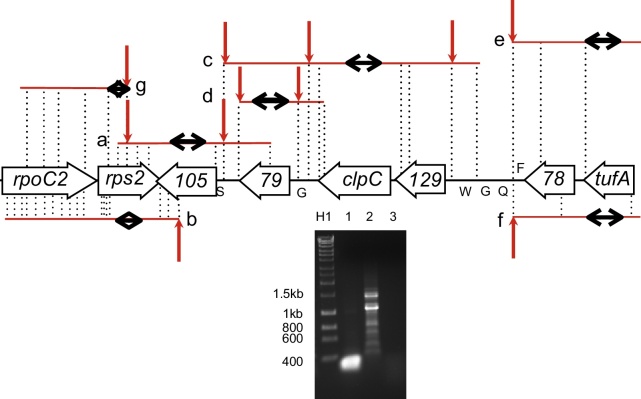
‘Transcription of *rps2*-*orf105*-*clpC*-*tufA*’ locus. Genes and transcripts are shown as Figure 2. Transcripts are shown above or below the genome depending on the DNA strand from which they were transcribed (note that sense and antisense are gene-specific, and genes are encoded on both DNA strands). Red arrows show major processing sites; further processing sites are indicated by dotted black vertical lines. The black arrows within each transcript indicates the region from which primers were designed. Not to scale, although processing sites shown to be conserved across multiple genes are aligned. The gel shows results of circular RT-PCR for *clpC.* Lane H1, hyperladder 1 kb (Bioline) with size markers indicated in bp, lane 1, internal (control) *clpC* PCR, lane 2 *clpC* circular PCR, lane 3 no RT-control *clpC* inwards PCR.

***rps2-orf105-clpC-tufA*** **Region.** We also studied the *orf105* region (Fig. 5). (Note that *orf105* has recently been renamed as *ycf93* (Goodman and McFadden 2014)). This gene is of particular interest as all genes upstream of *orf105* as far as the LSU rRNA are located on one DNA strand, with the exception of the gene for tRNA-Phe (UUC), while those downstream are located on the opposite DNA strand. The gene itself overlaps at the 5′ end with the *trnS* gene (24 bp) and at the 3′ end with *rps2* (in the opposite DNA strand, 14 bp). We performed circular RT-PCR and amplified products corresponding to transcripts of the *orf105* gene (Fig. 5, *transcript a*; Supplementary Material: supporting data S1). At the 5′ end of *orf105*, the majority of sequences recovered (21/28) represented transcripts with a 5′UTR of 61-64 nt, corresponding closely to the start of the *trnS* gene (at -62 nt). This site is shown with an arrow in Figure 5, *transcript a*. A minority (6/28) of cloned transcripts had a 5′ UTR between -7 (i.e. within the *orf105* gene) and +9 nt. The other transcript extended into *orf79*. At the 3′ end, 21/28 clones corresponded to transcripts ending at +105/106nt, with a further clone corresponding to a transcript ending at +101nt (as shown by an arrow in Fig. 5, *transcript a*). No transcripts extended as far as *rpoC2.* The results are consistent with a primary RNA molecule being cleaved upstream of *trnS* and also close to the 5′ end of *orf105* to give either *orf105* or tRNA-Ser, but not both (as the genes overlap). The strong 3′ terminus within *rps2* could represent either a transcription termination site, or a cleavage site.

Circular RT-PCR and amplification of cDNA corresponding to *rps2* (Fig. 5, *transcript b*; Supplementary Material: supporting data S1) gave rise to 39 clones. Of these, only 11 contained the full *rps2* coding sequence. The rest of the clones were missing the first 100-250 nt of *rps2* and did not have a specific 5′ end (Fig. 5, *transcript b*, labelled with dotted lines). However, the vast majority (32/39) of clones had a 3′ end at +175-177 nt (Fig. 5, *transcript b*, labelled with an arrow), corresponding to a transcript ending within *orf105* (which is on the opposite strand). No clones extended past this point, suggesting that it is a strong cleavage or transcription termination site.

We next carried out RNA circularization and PCR experiments with primers for the adjoining *clpC* and *orf79* genes (Fig. 5, *transcript c* and gel; Fig. 5, *transcript d*; Supplementary Material: supporting data S1). The majority (19/26) of *clpC* transcripts were polycistronic. The longest products, which also contain *orf79*, (8/26) ended immediately prior to *trnS*. This is consistent with the same cleavage site identified with *orf105* circularization (marked with an arrow in Fig. 5), suggesting it is a major processing site. In contrast, the majority (22/26) of *orf79* transcripts were monocistronic, and only 1 clone included both *orf79* and *clpC*. All the clones ended immediately following the stop codon of *orf79*, indicating a strong cleavage site. Together, these results would suggest that there is a long primary, polycistronic transcript which is first cleaved at *trnS*, followed by further processing to produce monocistronic *orf79*.

RNA circularization analysis for *tufA* (Fig. 5, *transcript e*; Supplementary Material: supporting data S1) revealed that 22/25 sequences recovered corresponded to transcripts ending at +315/317 nt, immediately at the start of the *trnQ* gene. The remaining three ended at 0, +7 and +214 nt from the 3′ end of *tufA*. No transcript extended into *trnQ*. There were 19 different 5′ end sites ranging from 0 to +739 nt.

### Antisense Transcripts

**Extensive polycistronic antisense transcription.** Antisense transcripts of the apicoplast genome have been reported for the apicomplexan *Toxoplasma*, but it is not known if they occur in *Plasmodium* as well. We therefore tested for the presence of antisense transcripts in the *Plasmodium* apicoplast. Note that in this section 5′ and 3′ are used in accordance with the associated gene (i.e. as if the transcript were transcribed from the sense strand).

**Figure 6:**
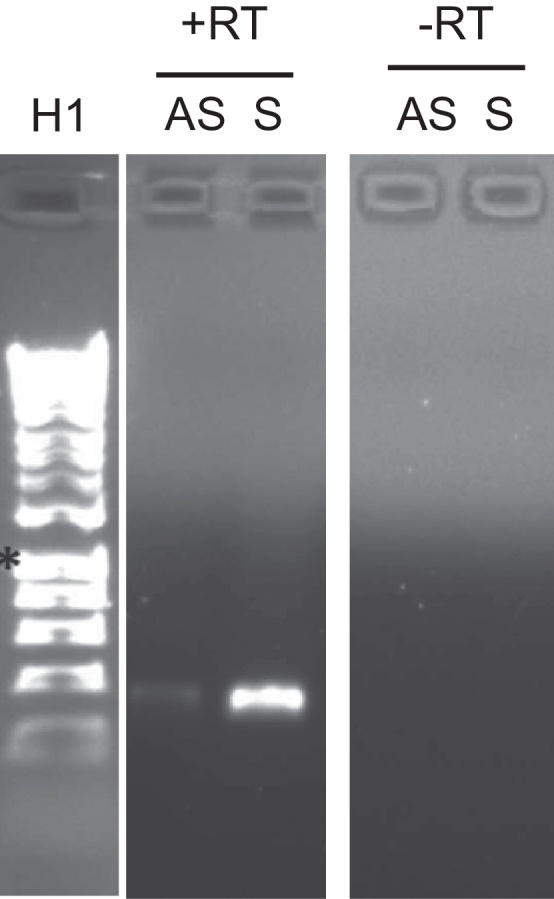
Sense and antisense transcription of *rps2*. Agarose gel showing transcription of both sense (S) and antisense (AS) transcripts from *rps2*, together with control reactions with no reverse transcriptase (RT). H1: hyperladder 1. All lanes are from one gel.

cDNA was synthesized using either a forward or a reverse primer (thus specific for sense or antisense transcripts) for eight genes (*rpoB, rpoC, clpC, tufA*, *orf105*, *SSU rRNA, rps2*, and *rpl2*), and this was used in PCR. For each gene, a band of the same size was seen for both sense and antisense transcripts when analysed by agarose gel electrophoresis. This indicates that both sense and antisense transcripts existed for each gene. Figure 6 shows results for *rps2*.

Next, we wished to determine if antisense transcripts could be polycistronic. We therefore synthesized cDNA using a forward primer at the extreme 3′ end of the LSU rRNA gene (the same locus as above), and then tested if it was possible to amplify sequences downstream of LSU rRNA from this cDNA by PCR. We successfully amplified a region from *trnE* to *rps19*. This indicating the existence of a long, polycistronic antisense transcript from LSU rRNA to *rps19* (Fig. 3).

We also tested the presence and extent of antisense transcripts for *sufB*. Analysis of transcripts containing the sense sequence of *sufB* had revealed that the gene is transcribed as a polycistronic molecule, extending at least as far as the adjacent *trnT* gene on the upstream side, and downstream through *orf51* and *orf101* into *rpoB* (Fig. 4, *transcript a*). In order to determine if there were also antisense transcripts spanning this region, cDNA was synthesized using a forward primer at the extreme 3′ end of the adjacent LSU rRNA gene. PCR was then carried out on the cDNA using the same forward primer but with reverse primers at intervals through the five genes (*trnT*, *sufB, orf51, orf101, rpoB*). Products of the expected size were obtained with all the reverse primers (data not shown), indicating that antisense transcripts extend from the 5′ end of LSU to at least 358 bp into *rpoB* (Fig. 4, *transcript b*). The antisense transcript thus covers at least four genes in full: *trnT, sufB, orf51* and *orf101*, as with the sense transcript previously identified.

**Processing of antisense transcripts.** Circular RT-PCRs to analyse sense strand transcripts had revealed that many transcripts are formed from the cleavage of polycistronic transcripts at processing sites associated with tRNA molecules. We therefore wished to determine if antisense processing occurs in the same way as sense transcript processing. Circular RT-PCR was therefore carried out to map antisense transcripts of *tufA, clpC, sufB, orf129, rpl16* and *rps2.* The same primer sets were used as before, except that cDNA was first synthesized with the forward primer (i.e. the opposite of that used in mapping sense transcripts). The majority of antisense circularization reactions did not give rise to corresponding recognizable product in the subsequent PCR. This was presumably because the levels of antisense RNA levels were too low, or because transcripts were too long to be identified using the RNA circularization technique. Products were obtained for *tufA, rps2* and *rpl2*, although success rates (in terms of clones obtained which contained recognizable apicoplast sequences) were much lower than for sense circularization experiments.

***tufA*** **antisense.** The circularization assay for antisense transcripts of *tufA* gave rise to six clones (Fig. 5, *transcript f*). At the 5′ end, one clone extended 123 nt upstream of the gene, four clones extended 78- 83 nt upstream, and one clone finished +8 nt (i.e. within the gene). None of these sites corresponded to the 19 different *tufA* sense cleavage sites previously observed. In contrast, at the 3′ end, five of the six antisense transcripts ended 315-317 nt after the end of the gene, as did 23/25 of the sense transcripts. This site maps immediately before the start of *trnQ*, suggesting that this is a major cleavage site for both sense and antisense transcripts.

***rps2*** **antisense.** The *rps2* and *orf105* genes are adjacent genes, but on opposing strands. They overlap by 14 bp at the 3′ end. Twenty eight clones generated from *rps2* antisense transcripts were identified and fully sequenced (Fig. 5, *transcript g*). None of the sequences covered the whole of the gene, and 19/28 clones corresponded to an antisense transcript with a 3′ end 118-121 nt before the 3′ end of the *rps2* gene (i.e. within the gene). This exactly corresponds to the 3′ end of the *orf105* sense transcript at +105-106nt (Fig. 5 *transcript a*; note that the gene is encoded in the opposing strand). Therefore, it seems likely that the majority of *rps2* antisense transcripts arise as a by-product from cleavage of a long, primary transcript containing the sense *orf105* mRNA.

### Cleavage of RNA

Analysis of the results from all circular RT-PCR experiments had indicated the presence of twelve major RNA processing sites (marked by arrows in Figure 3, Figure 4). Three sites are immediately adjacent to the 5′ or 3′ ends of individual genes (*SSU rRNA/ orf79)*, two sites are within the *rps2* and *orf105* genes, at the point where the orientation of genes on the apicoplast genome switches from one strand to the other. The remaining seven processing sites are immediately adjacent to tRNA sequences. The cleavage sites associated with the 3′ ends of *trnS, trnT, trnF, trnW, trnG* all coincided with the presence of an adjacent UUAU motif (UUAA for *trnG)*, Table 1. The cleavage site at the 5′ start of *trnG* also coincided with an associated UUAA motif, while the cleavage site associated with *rps2* was associated with a UUAG motif, while a UUAU motif was identified in *orf105.* No such motif was found near the *trnQ* cleavage site, which is conserved in both sense and antisense transcripts. These observations indicate that RNA cleavage is usually associated with a specific UUAA/U/G motif.

### RNA Editing

Alignments of sequence data revealed the presence of single point substitutions in individual transcripts. Although these could be caused by very low levels of RNA editing, we could not exclude that these were errors in reverse transcriptase, PCR or sequencing, and so did not analyse them further. However, an alignment of circularization data from *rpl2* with the corresponding genomic sequences revealed that 4/21 clones generated from circularized RNA indicated editing of a G to an A at position 649 within the gene, converting a glycine to a glutamate codon (clones marked with ** in Supplementary Material: supporting data S1). The clones were of different lengths, and therefore represent independent transcripts, making it unlikely that this event was caused by reverse transcriptase or PCR error. The clones containing the editing event were obtained from multiple independent circular RT-PCR experiments using RNA from different extractions (i.e. biological replicates).

To confirm the genomic sequence at this site, we amplified the region from genomic DNA by PCR, cloned the products and sequenced 20 clones. All clones contained a G residue, suggesting that the presence of an A in the RT-PCR products was indeed a result of RNA editing. When we carried out RT-PCR on linear RNA, we did not detect the editing site (0/9 clones and 0/13 clones from two different cDNA synthesis reactions). We next examined RNA-seq data from four *Plasmodium* libraries corresponding to four time points (10hr, 20hr, 30hr, 40hr; Siegel et al. 2014). This revealed that editing is stage specific, occurring only at 20 hours post-infection, as shown in Table 2. No evidence of editing was found at 10, 30 or 40 hours post-infection. Together these results suggest that RNA editing is stage-specific, and only occurs once RNA has been initially processed. This would account for the higher (25%) level of editing seen in the circularized RNA, which is only made up of processed RNA, over the lower (0-7%) levels observed in the RNA-seq data, which consists of both processed and un-processed RNA.

We next examined the *rpoC2* gene which encodes a subunit of the RNA polymerase (mis-annotated as *rpoD*, although it does not encode a sigma factor). This gene appears to contain a reading frame shift at position 1570-1575, where five A nucleotides encode either one or two lysine residues. No transcripts covering this region were identified in any of the four RNA-seq libraries. We therefore carried out RT-PCR across this region of *rpoC2* and cloned the PCR products into *E. coli*. All three sequenced clones contained the genomic version of *rpoC2* and none contained an edited version, suggesting that this gene is not edited despite the presence of a frame-shift mutation.

## Discussion

**Figure 7:**
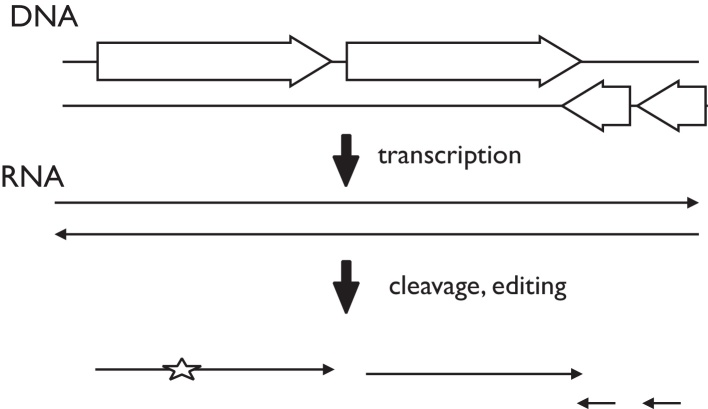
Summary of post-transcriptional processing. Schematic diagram. RNA editing event is indicated with a star.

We have shown that transcripts in the *Plasmodium* remnant chloroplast are polycistronic, confirming previous research (Figure 7) (Gardner et al., 1991a, Gardner et al., 1991b). The largest transcript we identified spanned 15 genes (Fig. 3). Circular RT-PCR indicated that transcripts covering individual genes had a range of different sizes. Where transcript ends were located within protein coding regions, the endpoints were generally heterogeneous, as with the 3′ ends of *sufB* transcripts, for example (Fig. 4). By contrast, many transcript ends coincided very precisely with the beginning or end of tRNA sequences, such as the 5′ ends of *sufB* transcripts, of which 95% coincided exactly with the start or end of the upstream *trnT* sequence. Similarly, 75% of *orf105* transcripts had a 5′ end corresponding to the start of the adjacent *trnS* sequence, and 88% of *tufA* transcripts extended through *orf78* with a 3′ end at the start of *trnQ*.

This suggests a processing pattern very similar to ‘Punctuation Processing’ first reported for human mitochondria, where polycistronic transcripts are predominantly cleaved by excision of tRNA sequences (Ojala et al. 1981). The generally heterogeneous location of ends within protein coding sequences may reflect non-specific processing, cleavage followed by exonucleolytic degradation, or non-specific transcription termination sites. The fact that transcripts cleaved at tRNA sequences have well-defined ends, even if they no longer retain the tRNA after cleavage, suggests that levels of artefactual exonucleolytic degradation are low in our assay.

There were two consistently observed instances of transcript ends within coding sequences. These were the 3′ ends of transcripts containing *rps2*, which were predominantly located close to a specific position within *orf105*, and the 3′ ends of transcripts containing *orf105*, which were predominantly located close to a specific position within *rps2*. These two genes mark the convergence of two long transcripts, with a transition from one genome strand being used for coding to the other. It is possible that the transcript ends correspond to specific transcript termination sites, although the antisense data (see below) suggest that at least some transcription can proceed through them. Major cleavage sites were associated with an UUAU motif. The mechanism of RNA cleavage remains to be elucidated.

It is striking that all regions of the genome tested were represented by antisense transcripts, although these were less abundant than sense transcripts, based on RT-PCR product levels. Although there have been previous reports of extensive antisense transcription of nuclear genes in *Plasmodium* (López-Barragán et al. 2011), we believe that this is the first evidence of antisense transcripts in the *Plasmodium* apicoplast. Antisense transcripts have previously been reported for the related apicomplexan, *Toxoplasma*, using a microarray tiling system, at 25 nt resolution, showing that the entire apicoplast genome is present on sense and antisense transcripts (Bahl et al. 2010).

Antisense transcripts could be generated either by direct antisense transcription or by read-through from a gene located on the opposite strand. The exact coincidence of the start of the antisense transcript of *rps2* and the 3′ end of the sense transcript of *orf105* suggests that the antisense *rps2* transcript may be generated by cleavage of a sense transcript of *orf105* extending into *rps2*. (Consistent with this, the existence of RNA molecules extending through the site could be detected by linear RT-PCR, data not shown.) Note that the different location of the 5′ end of the *rps2* antisense transcript from the 3′ end of the sense transcript confirms that the antisense transcript was not an artifactual amplification of the sense transcript. Many other antisense transcript processing sites corresponded closely to sense processing sites. For example, both *tufA* antisense and sense transcripts had a cleavage site corresponding with the start of the *trnW* gene. This may indicate that cleavage in these cases is primarily dependent on secondary structure rather than sequence (which will be different between the sense and antisense transcripts).

Whether antisense transcripts have a biological function in the apicoplast remains to be seen. It is known that the high levels of nuclear antisense transcripts seen in *Plasmodium* are stage-dependent, leading to speculation that these molecules could be involved in stage-specific regulation of gene expression (López-Barragán et al., 2011, Militello et al., 2005), and a similar process could be occuring in the apicoplast.

The occurrence of an RNA editing site in *rpl2*, altering the predicted amino acid sequence from a glycine to a glutamate, was unexpected. Our results indicate that RNA editing is stage-specific, and occurs after RNA has been cleaved into mRNA, as editing was observed only in cDNA derived from RNA which could be circularized in vitro (i.e. processed RNA) and not in cDNA derived from linear RNA, which includes RNA which has not yet been processed. If correct, this interpretation would also suggest that RNA which has not yet been processed constitutes a relatively large fraction of the RNA pool.

To our knowledge, RNA editing in the *Plasmodium* apicoplast has not previously been reported. In plant chloroplasts, RNA editing is restricted to C to U (or the inverse, U to C), and is generally uncommon. Some dinoflagellate algal species show extensive RNA editing, affecting around 5% of all nucleotides in *Karenia mikimoti*, although other species have very low (or absent) rates of editing, and the mechanism by which editing occurs is unknown (Barbrook et al., 2012, Dang and Green, 2010, Dorrell and Howe, 2012, Zauner et al., 2004). The edit seen here was G to A, which has been reported from dinoflagellates, although A to G editing is more common. Surprisingly, the editing site occurs in the only conserved region of *rpl2*, in the middle of a six amino acid consensus sequence (HPHGGG), as shown in Supplementary figure S2. It will be important to determine if this editing site occurs in other *Plasmodium* and/or Apicomplexan species.

No RNA editing was observed in *rpoC2*, despite the gene apparently requiring a frame-shift for translation. It is unclear how the frameshift is removed, though it is possible that this occurs during translation. Ribosomal frame-shifting in chloroplasts is not common; to our knowledge, the only report to date involves an artificially introduced *E. coli* gene in tobacco. Translation of this gene was successful in tobacco, suggesting that all the signals necessary for frame-shift were present in the gene, and that prokaryotic-style 70S ribosomes can carry out translation including a frameshift (Kohl and Bock 2009).

The occurrence of editing in the *Plasmodium* apicoplast is remarkable, as it has apparently been acquired independently of editing in dinoflagellates (given that some dinoflagellates, as well as *Chromera* and *Vitrella*, lack editing), and only a single site (or a few at most) is involved. It will be interesting to see if this is unique to *Plasmodium* or occurs in other parasitic Apicomplexa, such as *Toxoplasma*. The construction of a series of stage-specific RNA-seq libraries (enriched for apicoplast RNA, and not derived from polyA-tailed RNA) could help answer these questions.

Our results show that transcription and post-transcriptional processing in the remnant chloroplast of *Plasmodium* is complex. Some features, such as polycistronic transcription by a single RNA polymerase, appear to be conserved across *Plasmodium*, photosynthetic Apicomplexa, dinoflagellates and the red algae. Other features, such as RNA editing and ribosomal frameshifting, may be unique to specific lineages, and editing has apparently been acquired more than once. It is unclear whether RNA processing at tRNAs (Punctuation Processing) is an ancestral or derived characteristic, as very few tRNA genes have been identified in dinoflagellate chloroplasts (Barbrook et al. 2006), and little is known about how RNA is processed in red algae (from which the *Plasmodium* chloroplast ultimately derives). Punctuation processing is usually carried out by RNAseP, first identified in human mitochondria (Rossmanith et al. 1995) so if it is ancestral, a similar process is likely to be occurring in the *Plasmodium* apicoplast. A better understanding of red algal chloroplast RNA transcription and post-transcriptional processing would help resolve these issues. Nevertheless, the high level of post-transcriptional processing in *Plasmodium* offers important targets for the development of new antimalarial agents.

## Methods

***P. falciparum*** **culture:** Blood stage *P. falciparum* 3D7 was cultured according to Tarr et al. 2012 (Tarr et al., 2012). All work was carried out in accordance with the UK Human Tissue Act 2004. The apicoplast genome sequence is available on GenBank (accession numbers X95275 and X95276).

**RNA extraction:** Total RNA was extracted from *P. falciparum* according to Kyes et al. (2000). Asynchronous culture of at least 4% haematocrit was centrifuged at 800 *g* for five minutes and the supernatant removed. For every 300 μl of infected red blood cells, 5 ml Trizol (Invitrogen) was added. This mixture was incubated at 37 °C for five minutes with occasional shaking. One-fifth Trizol volume of chloroform (Sigma-Aldrich) was added with vigorous shaking, and left to stand at room temperature for three minutes before centrifugation at 1400 *g* at 4 °C for 30 minutes. Three-fifths Trizol volume isopropanol (Sigma-Aldrich) was added to the aqueous layer. The mixture was divided into 1.5 ml aliquots and each was spun at 16000 *g* at 4 °C for 30 minutes and the supernatant discarded. The glassy white pellet was resuspended in ice-cold 75% ethanol, centrifuged at 16000 *g* at 4 °C for 30 minutes, and the supernatant discarded. The pellet was resuspended in non-DEPC treated RNase free water (Invitrogen) and DNase treated (RQ1 RNase free DNase, Promega). RNA was purified using the Qiagen RNeasy mini spin column, eluted in RNase free water and stored at -80 °C until required.

**RNA circularization:** RNA circularization was carried out essentially according to Kuhn and Binder 2002. DNase treated RNA was circularized using T4 RNA ligase (Promega). Each reaction contained 300-750 μg RNA, 4 μl 10x T4 RNA ligase buffer, 0.5 μl recombinant RNasin (Promega), 1 μl T4 RNA ligase, 20 μl 40% w/v polyethylene glycol (Sigma), and RNAse-free water to 40 μl. The reaction was incubated at 37 °C or 42 °C for one hour, and then overnight at 16 °C. The circularized RNA was applied to an RNeasy mini column (Qiagen) and eluted in non-DEPC treated RNAse free water and stored at -80 °C until required.

**cDNA synthesis:** All primers are listed in the Supplementary Material, supporting Table S1. For each experiment, 1000-5000 ng RNA (either linear or circular) was used, to which was added 1 μl of 2 μM gene-specific reverse primer, 1 μL 10 mM dNTPs (Bioline) and non-DEPC treated water to 12.5 μl. The reaction was incubated at 65 °C for 5 minutes, and snap cooled on ice. 4 μl Superscript reverse transcriptase buffer (Invitrogen), 2 μl 0.1 M DTT, 0.5 μl recombinant RNasin (Promega) were added and the mixture incubated at 37 °C for 2 minutes prior to the addition of 1 μl Superscript II or III reverse transcriptase. The reaction was incubated at 37 or 42 °C for 50 minutes and the enzyme inactivated at 70 °C for 15 minutes. A no-RT control reaction was carried out for every cDNA synthesis reaction, where 1 μl dH_2_O was added in place of the Superscript reverse transcriptase.

RNA circularization experiments were performed in either duplicate or triplicate, from separate RNA preparations, and the data aggregated. All linear RT-PCR reactions were performed in triplicate, from separate RNA preparations.

**PCR, cloning and sequencing:** PCR was carried out on DNA, cDNA (or no-RT control) using GoTaq DNA polymerase (Promega). All primers were designed with an annealing temperature of 50 °C – 53 °C and all reactions were carried out with an extension temperature of 60 °C. Products were analysed by agarose gel electrophoresis, and sequenced where required. Where required, PCR products were cloned into pGEM-T-easy (Promega) and used to transform chemically competent *E. coli* TG1. Plasmids were sequenced using Sanger sequencing at the Depatment of Biochemistry, University of Cambridge sequencing facility.

**RNA-seq analysis:** Libraries containing transcriptome data for 10 hr, 20hr, 30hr and 40 hr post-infection were downloaded from the EBI. These correspond to study accession PRJEB3309, libraries ERR174301 (10 hr), ERR185969 (20 hr), ERR185970 (30 hr), ERR185971 (40 hr) sequences on an HiSeq2000 (Illumina) (Siegel et al. 2014). Sequences were aligned to the reference apicoplast genome sequence using the Bowtie2 plug-in in Geneious 8.0.5 (Kearse et al., 2012, Langmead and Salzberg, 2012).

## Figures and Tables

**Table 1:** **Sequences associated with major processing sites.** RNA sequences immediately adjacent to the predicted processing sites are shown for each gene. Processing sites are written in bold type and the adjacent UUAU/A motif is underlined. Note that *trnG* has three major processing sites, one 5′ and two 3′ to the tRNA.

**Gene**	**Sequence**
Ser (3′)	**U**UUAUAUU
Thr (3′)	**U**UUUAUUA
Trp (3′)	**U**UUUAUUA
Phe (3′)	**C**UUAUUAA
Gly (3′)	**A**UUUUAAA
rps2	U**U**AGAUUC
orf105	U**U**AUUUAA
Gly (5′)	UUAUAAAUUU**U**AA**C**

**Table 2:** **RNA editing in*****rpl2*****.** RNA-seq libraries from total RNA (poly(A)-tail enriched) were obtained from (Siegel et al. 2014). ‘Total’ refers to the total number of reads covering site 649 in the *rpl2* gene, and ‘Edited’ refers to the number of those reads that were edited G-->A.

	**10 hr**	**20 hr**	**30 hr**	**40 hr**
**Total**	151	104	674	482
**Edited**	0	7	0	0
**%**	0%	6.7%	0%	0%
