# Supplementary material for: Transcripts in the Plasmodium Apicoplast Undergo Cleavage at tRNAs and Editing, and Include Antisense Sequences
Source: Protist. 2016 Aug;167(4):377–88. doi: 10.1016/j.protis.2016.06.003 (PMC4995348; doi:10.1016/j.protis.2016.06.003)
Supplement: Supplementary file 1 [file mmc1.pdf]

Supplementary Data Nisbet et al.

Supporting Table S1 Primers used in this study

Primer name	Sequence
clpC circF	CCTTTATATGGAGCTCGT
clpC circR	CTGATATTTCTTTTAATTTTATTC
clpC intF	ACAGAAATATGGGTATTAAATGA
clpC intR	TGGAGTTGTAGGTAATATTAAAGG
orf79 circF	CTTGCTTATATTATTTATTATTA
orf79 circR	TTTCGTTTTATTTGATTTTGA
orf79 intF	ATTCAAAATCAAATAAAACGAAA
orf79 intR	TTTTGGATTATAAATACCTAA
orf105 circF	TGGAATGTATTATTAAATTATAA
orf105 circR	AAAAATATAAAAGAGAAAATGGG
orf105 intF	CCCATTTTCTCTTTTATATTTT
orf105 intR	TTATTTGAATTTTTACTTTTTAA
sufB circF	GTGGTTTAAGTATTTCAGAGTCTA
sufB circR	ATCATAATTTATATTTGGACAATC
sufB intF	GATTTTGCACAATTTGAACGTACT
sufB intR	CCACCATTACCTAAATAATCTCCTCTA
tufA circF	AATGTACAAAAAGTAGCTATACCT
tufA circR	CCCTATAGTACCTAAATTTATATG
tufA intF	ATATTGATTCAGCTCCAGAAGAA
tufA intR	TTCAATCTTACCTGTTACTACTG
SSU circF	CGTAACAAGGTAGCCGTACTG
SSU circR	TTAATTCTGAGCTAGGATCAAA
SSU intF	CTTGTATAAGCTCCCGTCAA
SSU intR	AGGAGCATAATGACTTGACG
rps2 circF	TCTACCTAAATACATATTTTTAACA
rps2 circR	CCAGAAACCCATTTATTTA
rps2 intF	GGGTTTCTGGATTATTAACT
rps2 intR	TGTTAAAAATATGTATTTAGGTAGA
rpl2 circF	AAATGTATGTGACCATCCTC
rpl2 circR	ATTTCCACCTCCTTTATTATAA
rpl2 intF	GATCAGCAGGTACTTTTGCT
rpl2 intR	CATGAGGATGGTCACATACA
rpl2 editR	CGACCAATACCAGTCTTTCC
LSU F	GTGTATAATTCCTAATAAGTTGA
rps4 R	ATAATACCTTGATTGCAATATAG
tRNA-Tyr R	CAGATTTGAACTGATGTAGATA
tRNA-Asp R	CGAGAATTGAACTCGTATTC
tRNA-Glu R	TCGAATTCCTATTTTCTTCTTG
tRNA-Glu F	CAAGAAGAAAATAGGAATTCGA
rpl4 R	CCTAGCCCTTTTTGTACTC
Rpl23 R	AAAAATTTAAAATAACTTCTTTCA
Rps19 R	AAATAGGTATAAATTTATATCCAT
tRNA Val F	GCTTTACATACAGAAGACCAT
SSU R	TTAATTCTGAGCTAGGATCAAA
LSU F	GTGTATAATTCCTAATAAGTTGA
tRNA-Val F	GCTTTACATACAGAAGACCAT
rpoB R	TACTAAAATAGGATTAACAATATA

Supplementary Figure 1

Extract from Genbank accession X95275.2 *Plasmodium falciparum* complete gene map for plastid-like DNA (IR-A) (i.e. apicoplast genome sequence)

```
4981 tagtaatata tttagtgtat aattcctaat aagttgaata tttaattatt tattgttata
5041 tttgctaaag tagcttaatt ggtaaagcaa ctgatttgta atcagtagat tatgagttca
5101 aatctcacca ttagctttta ttatttttat atataattat tatgataaaa ttaaaaaatt
5161 ttttaaatat ttataattta aattataaat atcaatataa aaataaaata aatttatatt
5221 taataagaca aggattaaat ataaatttaa taaaaaattt atctagtaat attttttat
5281 atatgtttat ttataatttt aaaaaatatt ctttaaaatt attaaatata tttaaattac
5341 ctgattggaa tttttttgat tgtccaaata taaattatga taatattatt tattattctt
5401 ctattttaaa agataataat ttaatatatt atttaaaaaa taatttaaat attgaatttt
```

Sequence highlighted in purple encode *tRNA-Thr*
Sequence highlighted in yellow encode *sufB* (partial, 5' region only)
Numbers refer to full accession.

The two bold residues **g** (position 5044) and **t** (position 5116) are the two cleavage sites identified in *sufB* circular RT-PCR experiments, exactly coinciding with the *tRNA-Thr* gene. The adjacent TTAT sites are underlined. Below is a tRNA-SE scan (Schattner, 2005) predicted structure of the tRNA (in black). The adjacent non-coding sequence is shown in blue, and the UUAU motif in red.

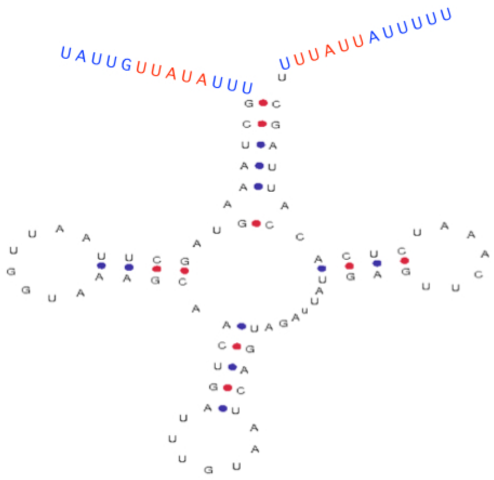

Reference:
Schattner, P., Brooks, A.N., and Lowe, T.M. (2005) Nucleic Acids Res, 33: W686-689.

Supporting Data S1

Results from circular RT-PCR for eight genes. Negative numbers refer to residues within a gene (i.e. transcripts that terminate within a gene). For rpl2 only, a ** following the clone number indicates the presence of an RNA editing event.

SSU- 1426bp

Sense Clone	Residues before 5' start	Residues after 3' end	Total transcript length
1	46	148	1620
2	46	148	1620
3	-1	-6	1419
4	-1	-6	1419
5	-1	-6	1419
6	-1	-7	1418
7	-1	-7	1418
8	-1	-7	1418

sufB -1413 bp

Sense Clone	Residues before 5' start	Residues after 3' end	Total transcript length
1	98	1199	2710
2	98	746	2257
3	98	596	2107
4	98	596	2107
5	98	510	2021
6	98	399	1910
7	98	374	1885
8	98	360	1871
9	98	360	1871
10	98	360	1871
11	98	305	1816
12	98	251	1762
13	98	248	1759
14	98	248	1759
15	98	246	1757
16	98	231	1742
17	98	189	1700
18	104	97	1614
19	98	90	1601
20	25	138	1576
21	25	134	1572
22	25	29	1467
23	25	25	1463
24	25	25	1463

Antisense Clone	Residues before 5' start	Residues after 3' end	Total transcript length
1			738
2			738
3			738
4			738
5			738
6			738
7			738
8			738
9			738
10			738
11			738
12			738
13			738

orf105 -366bp

Sense Clone	Residues before 5' start	Residues after 3' end	Total transcript length
1	240	105	711
2	61	141	568
3	64	134	564
4	62	106	534
5	62	106	534
6	62	106	534
7	62	106	534
8	62	106	534
10	62	106	534
11	62	106	534
12	62	106	534
13	62	106	534
14	62	105	533
15	62	105	533
20	62	105	533
16	62	105	533
17	62	105	533
18	62	105	533
19	63	77	506
20	62	58	486
21	9	105	480
22	3	105	474
23	62	44	472
24	0	105	471
25	0	105	471
26	0	101	467
27	-5	105	466
28	62	30	458

orf79 - 240bp

Sense Clone	Residues before 5' start	Residues after 3' end	Total transcript length
1	187	0	427
2	104	1	345
3	103	0	343
4	103	0	343
5	26	0	266
6	26	0	266
7	26	0	266
8	26	0	266
9	26	0	266
10	26	0	266
11	26	0	266
12	26	0	266
13	26	0	266
14	26	0	266
15	26	0	266
16	26	0	266
17	26	0	266
18	26	0	266
19	26	0	266
20	26	0	266
21	26	0	266
22	26	0	266
23	26	0	266
24	26	0	266
25	26	0	266
26	26	0	266

clpC -2301 bp

Sense Clone	Residues before 5' start	Residues after 3' end	Total transcript length
1	481	348	3130
2	398	347	3046
3	398	347	3046
4	398	347	3046
5	398	347	3046
6	398	347	3046
7	398	347	3046
8	398	10	2709
9	398	10	2709
10	398	10	2709
11	398	10	2709
12	398	10	2709
13	398	10	2709
14	398	10	2709
15	398	10	2709
16	398	10	2709
17	398	10	2709
18	398	10	2709
19	127	10	2438
20	10	10	2321
21	10	10	2321
22	10	10	2321
23	10	10	2321
24	10	10	2321
25	-146	0	2155

rps2- 684bp

Sense Clone	Residues before 5' start	Residues after 3' end	Total transcript length
1	721	127	1532
2	661	176	1521
3	470	177	1331
4	456	176	1316
5	307	176	1167
6	260	177	1121
7	228	177	1089
8	118	177	979
9	96	177	957
10	56	177	917
11	172	34	890
12	323	-120	887
13	-73	176	787
14	-140	176	720
15	-139	177	722
16	-137	177	724
17	-154	177	707
18	-156	176	704
19	-177	177	684
20	-193	176	667
21	-197	177	664
22	-212	177	649
23	-215	176	645
24	-224	177	637
25	-224	176	636
26	-224	176	636
27	-224	176	636
28	-228	175	631
29	-239	177	622
30	-256	175	603
31	-261	177	600
32	-261	177	600
33	-266	177	595
34	-266	177	595
35	-266	176	594
36	-190	71	565
37	-12	-120	552
38	-256	-115	313
39	-256	-115	313

Antisense Clone	Residues before 5' start	Residues after 3' end	Total transcript length
1	476	-189	971
2	382	-121	945
3	201	-118	767
4	54	-119	619
5	-33	-119	532
6	-166	-121	397
7	-155	-155	374
8	-237	-119	328
9	-246	-119	319
10	-246	-119	319
11	-250	-119	315
12	-252	-118	314
13	-252	-118	314
14	-255	-119	310
15	-255	-119	310
16	-255	-119	310
17	-186	-189	309
18	-186	-189	309
19	-186	-189	309
20	-186	-189	309
21	-186	-189	309
22	-269	-120	295
23	-269	-120	295
24	-269	-120	295
25	-269	-120	295
26	-269	-120	295
27	-205	-190	289
28	-229	-179	276

tufA-1233bp

Sense Clone	Residues before 5' start	Residues after 3' end	Total transcript length
1	739	315	2287
2	707	317	2437
3	652	317	2382
4	521	316	2250
5	290	317	2020
6	247	317	1977
7	212	316	1941
8	197	317	1927
9	197	317	1927
10	192	317	1922
11	182	317	1912
12	101	317	1831
13	101	317	1831
14	75	318	1806
15	58	315	1786
16	51	317	1781
17	51	317	1781
18	51	317	1781
19	42	316	1771
20	42	316	1771
21	20	317	1750
22	20	317	1750
23	19	214	1646
24	63	0	1476
25	0	7	1420

Antisense Clone	Residues before 5' start	Residues after 3' end	Total transcript length
1	123	317	1673
2	83	317	1633
3	78	317	1628
4	78	317	1628
5	78	315	1626
6	-8	124	1349

rpl2 - 738bp

Sense Clone	Residues before 5' start	Residues after 3' end	Total transcript length
1 **	899	178	1815
2	899	127	1764
3	368	563	1669
4 **	551	296	1585
5	387	434	1559
6	74	570	1382
7	56	502	1296
8	56	502	1296
9	50	439	1227
10	127	302	1167
11	212	111	1061
12	212	111	1061
13 **	156	165	1059
14	20	268	1026
15	123	17	878
16 **	-52	311	997
17	-52	311	997
18	-23	275	990
19	-64	314	988
20	-11	150	877
21	*-49*	*-5*	684
