# Supplementary material for: Transcripts in the Plasmodium Apicoplast Undergo Cleavage at tRNAs and Editing, and Include Antisense Sequences
Source: Protist. 2016 Aug;167(4):377–88. doi: 10.1016/j.protis.2016.06.003 (PMC4995348; doi:10.1016/j.protis.2016.06.003)
Supplement: Supplementary file 2 [file mmc2.pdf]

```
P. tetraurelia   RTEKP-ILKAGRQFHKYRRLRKCWPKVRGVAMNPVDHPHGGGNQQ-HIGHPSTLSRYAPP
C. velia         NHKFCRLGKAG-----LNILKHKKPTVRGTAMNAVDHPHGGGTGKAGIGRTAPLTPWGKL
P. marinus       NFWARAKGHCR-----VKRWLGWRPSVHGVAKNPVEHPHGGGTSHKGPKRN-PVSPWGGL
P. infestans     NHKKIKLGKAG-----RSRWLNRRPSVRGVAKNPVDHPHGGGEGKTSGGRP-SVTPQGKI
K. venefium      EYKNLVIGKAG-----RNRWFGKRPKVRGLAKNPNDHPHGGGEGRCGIGRT-PSTPWGKP
P. tricornitum   DAFLVQSGKAG-----RTRWLGKRPTVRGSVMNPCDHPHGGGEGRAPIGRTRPLTPWGKP
E. huxleyi       DAINTCLGKAG-----RSRWLGKRPKVRGVVKNPIDHPHGGGEGRSPIGRAKPVTPWGQP
B. bovis         KIEEPKYKTAG-----YKIKLGRRPKVRGTAMNACDHPHGGGEGKAPIGRKTIYSFTGRK
L. caulleryi     YYNKYKIKNAG-----YNRYYNKRPKVRGKAMNACDHPHGGGKGKTSIGRKYPCSKKGLH
P. falciparum    FHNKFKIKNAG-----YNIYYNIKPKVRGKAKNVCDHPHGGGKGKTGIGRKYPCSKKGLH
P. chibaudi      LYNKFKIKNSG-----YNIFYNKRPKVRGKAKNVCDHPHGGGKGKTSIGRKYPCSKKGLH
P. berghei       LYNKFKIKNAG-----YNVFYNKKPKVRGKAKNVCDHPHGGGKGKTGIGRKYPCSKKGLN
P. yoeilli       LYNKFKIKNAG-----YNVFYNKKPKVRGKAKNVCDHPHGGGKGKTGIGRKYPCSKKGLH
P. malariae      LYNKFKIKNAG-----YNIYYNIKSKVRGKAMNTCDHPHGGGKGKTSIGRKYPCSKKGLH
P. gallinacuem   FYNKYKIKNAG-----YNIYYNKKSHVRGKAMNVCDHPHGGGKGKTSIGRKYPCSKKGLH
P. ovale         LYNKFKIKNAG-----YNMYYNIKSKVRGKAKNVCDHPHGGGKGKTSIGRKYPCSKKGLH
P. vivax         LYNKFKIKNAG-----YNIYYNIKSKVRGKAKNVCDHPHGGGKGKTSIGRKYPCSRKGLH
P. cynemolgi     LYNKFKIKNAG-----YNIYYNIKSKVRGKSKNVCDHPHGGGKGKTSIGRKYPCSKKGLH
P. coatmeyi      LYNKFKIKNAG-----YNIYYNIKSKVRGKSKNVCDHPHGGGKGKTSIGRKYPCSRKGLH
T. gondii        KVNKNIKKHAG-----FNRKLSIRPKIRGAAMNAVDHPHGGGEGKASVGFKFARTLWGKA
E. tunella       LFLK--KNKAG-----FNRLLGKRPKVRGVAMNACDHPHGGGEGKNSIGRSSVYSPWGTI
                          .                ::*   *  :******  :         :  .
```

Figure S2 Alignment of amino acid sequences of the region surrounding the *rpl2* edit site in selected alveolate species using Clustal Omega alignment software. Conserved sequences are marked as an asterisk at the bottom of the alignment. The edited site is highlighted in yellow.
